# Findings and Recommendations from the NIST Workshop on Alternative Fuels and Materials: Biocorrosion

**DOI:** 10.6028/jres.120.003

**Published:** 2015-03-11

**Authors:** Elisabeth Mansfield, Jeffrey W. Sowards, Wendy J. Crookes-Goodson

**Affiliations:** National Institute of Standards and Technology, Boulder, CO 80305; Soft Matter Materials Branch, Materials and Manufacturing Directorate, Air Force Research Laboratory, Wright-Patterson AFB, OH 45433

**Keywords:** alternative fuels, bacterial, biofilm, biosusceptibility, corrosion, microbial

## Abstract

In 2013, the Applied Chemicals and Materials Division of the National Institute of Standards and Technology (NIST) hosted a workshop to identify and prioritize research needs in the area of biocorrosion. Materials used to store and distribute alternative fuels have experienced an increase in corrosion due to the unique conditions caused by the presence of microbes and the chemistry of biofuels and biofuel precursors. Participants in this workshop, including experts from the microbiological, fuel, and materials communities, delved into the unique materials and chemical challenges that occur with production, transport, and storage of alternative fuels. Discussions focused on specific problems including: a) the changing composition of “drop-in” fuels and the impact of that composition on materials; b) the influence of microbial populations on corrosion and fuel quality; and c) state-of-the-art measurement technologies for monitoring material degradation and biofilm formation.

## 1. Workshop Summary

An open-registration workshop was hosted by NIST in Boulder, CO on July 22–23, 2013. The purpose of the workshop was to identify and explore the unique materials and chemical challenges that occur with manufacturing, transport, and storage of alternative fuels. Specific foci of the workshop were:

- The changing composition of fuels and its influence on materials for transportation and storage infrastructure;
- The influence of microbial populations on corrosion; and
- State-of-the-art measurement technologies for monitoring material breakdown and biofilm formation.

Seventy-five attendees were present in the workshop representing various government agencies, academic institutions, and industry. The workshop agenda is shown in Table 1. The workshop included discussions of various topics, and also completion of an attendee survey shown in Table 2. The workshop discussion and general results of the survey are presented in the report below.

### Statement of the problem

The cost of corrosion is estimated at $2.2 trillion according to the World Corrosion Organization [1]. Based on the estimate that 20 % of all corrosion is attributable to microbiologically influenced corrosion, or MIC [2–4], the cost of MIC could be on the order of $440 billion.

Microbiologically influenced corrosion (MIC) is generally defined as corrosion that occurs as a result of microbial activity. MIC-causing microorganisms, such as bacteria and fungi, often form biofilms on materials, inducing chemical changes in the material and in the surrounding liquid medium. Some common bacteria found to be associated with engineered materials in alternative fuel systems include acid-producing bacteria, sulfate-reducing bacteria, and iron-reducing bacteria [2,3,5]. These bacteria enable MIC through a variety of mechanisms, including: increasing the corrosion potential of metal alloys, generating acid, changing oxygen concentrations, and by directly oxidizing or reducing elements in engineering alloys. These chemical changes can lead to localized attack, resulting in pitting and dealloying, and can also exacerbate erosion corrosion, stress corrosion cracking, and hydrogen embrittlement [5,6]. While MIC is distinct from corrosion that is not influenced by microbes, the fundamental mechanisms are often similar to non-microbial corrosion. Thus, indicators that lead towards specifically diagnosing corrosion as MIC are poorly defined. MIC of alternative fuel systems is an extraordinarily complex problem, dependent on many conditions, including microbe presence, type and activity; material type and composition; and fuel chemistry and the susceptibility of the fuel system to biocontamination. Because of the associated complexities, MIC is often used as a “catch-all” term to explain hard-to-understand corrosion cases after all other theories have been discarded [7]. Furthermore, a clear understanding of the initiation and mechanism of MIC does not exist since fundamental research of MIC lags behind applied research, which is only aimed at diagnosing or solving a particular problem.

The corrosion and MIC research communities lack an accurate estimation of the occurrence and prevalence of MIC to fully predict infrastructure reliability. Often general statements are made, such as “20 % of total corrosion is attributable to MIC”, but these numbers are often quoted without certainty of their accuracy and can vary widely based on their source [2–4]. There is a lack of consensus on the frequency of MIC in the United States and worldwide, which raises significant questions regarding the far-reaching nature of the problem and the possibility that many more cases exist than those that are reported. MIC may be underestimated for various reasons including: non-disclosure by industries or government agencies, non-reporting by maintainers who consider it a ‘hygiene issue’ and do not want negative attention or fear repercussions from regulatory agencies, lack of resources or qualified personnel to diagnose properly, and lack of distinguishing MIC characteristics that prevent concrete identification.

In general, responses to microbiologically influenced corrosion tend to be more reactive than proactive. There is significant evidence that diagnosis of MIC is not universal or timely, resulting in widespread incidents or failures that may eventually be attributed to MIC. Much of the data documented as MIC-related failures or incidents comes from anecdotal stories from the field, is restricted from release by trade secrets or regulation, or is presented as somewhat skeptical results obtained from non-standardized testing methods. Significant infrastructure failures resulting from MIC have been reported (e.g., Alaska pipeline failure in 2006) [8], but smaller (i.e., less costly, smaller volume, or less publicized) incidents may not necessarily be reported. Microbial mitigation strategies (e.g., biocides) are often only put into place after significant problems are observed and other mitigation techniques have been ineffective.

There are no *definitive* tests for MIC, so the current process used when MIC is suspected is lengthy and expensive. Many of the microbial species that are associated with MIC are common throughout nature and are often found in environments that are not associated with corrosion; thus, detection of their presence in an environment in which corrosion is observed does not necessarily mean they play a role in the corrosion mechanism. Diagnosing MIC requires extensive field and laboratory analyses that may take three to six months to complete [7,9]. In addition, there is a strong tendency to require other mechanisms of corrosion to be disproved before MIC is diagnosed and mitigated, which lengthens the process [2]. This diagnosis process is intractable and too slow for many industries and agencies, which need to react and mitigate faster than is currently possible. A paradigm shift in MIC root cause analysis is required. Such a paradigm shift may be enabled by an increase in data exchange, the ability to extract new information from shared datasets, and by additional research.

There is also a stigma associated with reporting cases in which microbes are thought to have played a role. Data from industry on MIC failures is inaccessible to the larger MIC research community. Corporations and contractors who mitigate MIC protect trade secrets, customers, and stakeholders, never releasing reports to the broader community, and restrictions on data release from U.S. government agencies limit a broad understanding of the problem. In addition, the major experts with knowledge relevant to MIC (including microbiologists, chemists, inspectors, regulators, metallurgists) belong to diverse scientific communities, with little interaction. Data sharing from those outside the MIC research community, which may include potential cross-over mitigation strategies from medical biofilm researchers, is rare.

The inability to fully comprehend the magnitude of MIC, and diagnose it quickly, has serious implications on the maintenance of infrastructure, and by extension, on safety and national security. MIC can directly impact safety by causing pipeline failures and the subsequent release of fuel contaminants into the environment [4]. Beyond protection of the environment and fuel infrastructure, determining the impact of environmental perturbations of the system, intended or unintended, is impossible. For example, because we have a poor understanding of what level of MIC may always be present (a “baseline” level of MIC), we cannot predict or determine the impact of natural disasters, such as hurricanes, where structures previously not exposed to MIC-friendly conditions are suddenly exposed. Predicting impacts when industries/agencies implement greener, more microbe-friendly materials or when we continue to use aging infrastructure past its lifetime, is also impossible at this time. Thus, understanding the pervasiveness of MIC, and improving our ability to diagnose it, would allow us to better anticipate and respond to potential safety and security issues as they occur.

## 2. Preliminary Research Priorities

There is an opportunity to develop a greater understanding of MIC mechanisms by defining the prevalence of the problem, working towards gathering the community together, and leading more fundamental research efforts. These efforts are broken down into the major topic areas of fuels, infrastructure materials, and microbiology.

### 2.1 Fuels

#### 2.1.1 Defining Biosusceptibility

At this point, there are few predictive measures in place that allow systematic evaluation of the susceptibility of emerging fuels to microbial contamination or evaluation of their ability to contribute to corrosion [10]. The identification of the molecular structures of individual fuel components that show increased risk for microbial degradation needs to be accomplished [11]. Fuel components are widely investigated for their compatibility with a given fuel system. Fuel additives, for example, are often investigated for interactions with materials, such as their ability to form deposits on engines, among other tests [12]. Similar evaluations need to be employed in systems where biologically-derived fuels are used or could be used, and where there is increased risk of biocontamination. Biodiesel fuel, for example, is highly susceptible to microbial contamination due to its high oxygen, water, and fatty acid methyl ester content, all of which encourage microbial growth. In addition, biodiesel stocks are often contaminated with glycerol, which can be utilized by bacteria to form other corrosive or detrimental products [13]. Not only would secondary metabolic compounds be a problem, but fouling of the fuel system and changing fuel properties due to the addition/removal of specific compounds would be expected. These effects may be detrimental to fuel transportation and storage infrastructure or fuel quality. There is a need to develop a “biosusceptibility index” for a fuel. In theory, this biosusceptibility index could allow for predictive properties for new “drop-in” fuel types, as well as new cost-saving measures to predict appropriate intervals for adding biocide.

#### 2.1.2 Fundamental Properties

The key to understanding, and therefore predicting, the properties of gaseous and liquid fuels is through the fundamental properties that can be linked solidly with theory. This approach has succeeded with thermodynamic and transport properties, but it is apparent that the applicability to the complex compositional changes that might occur because of fuel biocontamination and MIC will require major advances. Both biocontamination and MIC have the potential to change fuel composition through the addition of bacterial or metallic ion content (as corrosion occurs), and also through the processing of fuel components into secondary products that would not normally be present in the fuel. These changes in fuel composition can have a direct impact on fuel properties. To predict the impact on the fuels, first, we must develop indicators of microbial contamination, and second, determine the effects of such contamination, from both an analytical and fluid property standpoint. This is a departure from traditional coverage of fluid properties because (1) MIC has the potential to cause production of compounds with very different chemical properties (e.g., polarity, polarizability, solvation) than typical fuel constituents, and (2) even trace quantities of these very different constituents can drastically affect fuel properties. Thus, no fundamental description of fuel properties, as influenced by biocontamination and MIC, will be complete without explicit consideration of these aspects. Moreover, it is not currently possible to theoretically model the properties of mixtures of highly polar constituents with nonpolar constituents. This would be necessary for understanding the impact of how fuel can be impacted by biocontamination and MIC.

### 2.2 Microbiology

#### 2.2.1 Presence versus Impact

The introduction of microbial identification tools, particularly non-cultivation-based molecular tools such as quantitative real time polymerase chain reaction (PCR) and high throughput 16S/18S ribosomal DNA sequencing, is considered to be a significant advance in understanding the role of microbes in MIC [14]. In many systems and use conditions, identification of the presence of microbes through use of these identification tools is satisfactory. However, merely knowing ‘who is there’ is not sufficient to understand the role of microbes in MIC, as the activity of the microbe within the system is more important than its mere presence. Mechanistic modeling is needed to identify the role microbes play in MIC. Understanding the metabolic processes that govern the growth and persistence of microorganisms in fuel environments and/or in contact with infrastructure materials will allow for the development of effective mitigation strategies through the control of metabolic processes or inhibition of MIC causative agents. There are classes of microbes that are known to have a significant impact (e.g., acid-producers, sulfate-reducers) on the materials in alternative fuel systems but the relationship between these classes and other microorganisms in a contaminated fuel system is poorly understood. Mathematical models could lead the way towards predicting the impact and need for biocide deployment. Models also are beginning to show promise in their ability to demonstrate mechanical and chemical interactions between different microbes present in complex biofilms. The interactions are a problem that has long been difficult to measure.

### 2.3 Infrastructure Materials

#### 2.3.1 Detection and Monitoring

Better measurement tools to detect MIC and monitor mitigation for MIC damage in pipelines, storage tanks and other relevant alternative fuel systems are needed. MIC in alternative fuel environments is an emerging area, and there is potential for regulatory government agencies (e.g., Department of Transportation, Environmental Protection Agency in the U.S.) to directly feed field inspection data into a database maintained by a neutral third party interested in standardization, such as the National Institute of Standards and Technology, allowing NIST to trace the impact of detection and monitoring of MIC. The detection and monitoring data could be used to support development of methods that would allow for better prediction, as much of the community utilizes different strategies for collection of MIC-relevant data. Detection and monitoring systems are currently the most critically needed tools in the field of MIC, since response to incidents as a result of failures can often outweigh the efforts in prevention.

#### 2.3.2 Prediction

There is not currently a repository of metallurgical data that indicates what would be observed in a MIC failure versus other corrosion mechanisms. There is an opportunity to develop metallurgical metadata consolidation practices to match experimental metallurgy results with more specifics about what contributed to the corrosion. One of the major problems in identification of MIC is that often, the whole story is not being told; metallurgists don’t always consider or explain biological information and biologists don’t consider metallurgical changes. Gathering relevant data from all aspects (fuels, microbes and materials) would help identify potential MIC markers. By developing partnerships with relevant industries, a non-regulatory agency, such as NIST, may be able to gather field data that would allow for predictive modeling of MIC situations. Through the development of sample collection and monitoring strategies that help support the data leading towards predictions, NIST would position itself to develop predictive capabilities for any future transport and storage needs, especially since the number of alternative fuels is rapidly increasing.

### 3.1 Standardization and Community Engagement

#### 3.1.1 Standardization

MIC researchers can play an active role in standards development leading towards comprehensive understanding of MIC mechanisms. A more prominent presence by researchers in standards organizations that are producing MIC-related standards, such as ISO, ASTM and NACE, is needed to lead efforts in standardization of relevant protocols (such as standards for sample preservation) that enable fundamental understanding of MIC. Through these organizations, organizations such as NIST can lead efforts in the identification of MIC-relevant mechanisms and provide fundamental research that guides the community towards understanding. Unified efforts to provide physical standards, such as standard corrosion test coupons and a biocorrosion-relevant standard microbial community are needed. These physical standards could be utilized to perform interlaboratory studies and feed into databases that enable understanding of what distinguishes MIC from other types of corrosion.

#### 3.1.2 NIST Role in Engaging the Community

NIST organized the workshop to discuss the problem of MIC in alternative fuel systems with the multidisciplinary community. The workshop participants further identified a unique opportunity for NIST to overcome problems associated with data exchange by acting as a neutral, non-regulatory party. Increased data sharing is needed in a way that allows for the release of valuable data towards understanding, but in a way that removes all identifying features. Only through a mechanism such as this could industries and agencies report data that would enable significant advances in MIC understanding without suffering repercussions or releasing trade secrets/problems. Other NIST efforts to collect, analyze, and distribute very large datasets could be a useful analysis tool for the MIC research community. This database could be used by researchers, modelers, and statisticians, to provide fundamental links between observed problems, and to work towards a fundamental understanding of material failures.

## 4. Research Priorities

In summary, the extent of corrosion attributable to MIC and the potential impact of the problem are ill-defined. While the community believes microbiologically influenced corrosion exists, there are not clear, irrefutable markers that delineate MIC as a mechanism over other types of corrosion. Since 1985 when the National Bureau of Standards held a workshop on MIC in conjunction with the National Association of Corrosion Engineers [15], the need to delineate what corrosion is microbiological in nature has not changed.

As a result of the discussions during the workshop, research priorities were identified. These priorities are outlined here:

- *Metallurgical changes need to be attributed to microbial activity*. Research should focus on what changes microbes make in metals and their alloys. Advanced tools are needed to identify these metallurgical markers in the field. The main priority of this work is to identify if the corrosion observed is attributable to microbes or to another cause.
- *The impact of microbe activity needs to be investigated*. Microbes will be present in the environment and identification of microbes can be important in initial stages, but the real focus of the research in this area should be aimed at determining if the microbes present are active or inactive. In addition, complex systems with multiple microbiological species should be investigated to understand how these systems can support and enable one another.
- *Development of standards is crucial*. Test methods are needed to ensure that accounts of microbiologically influenced corrosion are being cataloged correctly. Sampling of corrosion in the field, especially with a focus on preservation of microbiological samples, should be a top priority for standards development.
- *Fuel chemistry will become increasingly important*. The changing climate of fuel chemistry, particularly with the implementation of “drop-in” fuels, will have an impact on MIC. Emerging biofuels may be more susceptible to degradation or processing by microbes, and special attention should be aimed at ensuring that fuel chemistry changes are investigated alongside metallurgical and biochemical systems important for MIC.

## 5. Conclusions

The 2013 Applied Chemicals and Materials Division workshop on Alternative Fuels and Materials: Biocorrosion was held to identify and explore the unique materials and chemical challenges that occur with manufacturing, transport, and storage of alternative fuels. Through participation by industry, government, and academia in the areas of fuels, microbiology and infrastructure materials, research priorities were identified. There was a clear consensus that researchers from all fields need to work together to prioritize detection and identification of corrosion suspected to be MIC related. If the research priorities identified in this workshop were addressed, the cost of corrosion could be dramatically reduced and infrastructure changes could be planned to accommodate fuel chemistry as we enter an era of renewable biofuels.

## Figures and Tables

**Table 1:** Workshop agenda

**Workshop on Alternative Fuels and Materials: Biocorrosion**
Organized by the Applied Chemicals and Materials Division
National Institute of Standards and Technology
July 22 – 23, 2013
325 Broadway, Boulder, CO 80305
**Day 1**
Welcome
*Elisabeth Mansfield, National Institute of Standards and Technology*
Keynote: Biocorrosion: How do we know?
*Speaker: Gary Jenneman, ConocoPhillips*
Keynote: Biocorrosion in the Diesel Fuel Infrastructure: Impact of Ultra Low Sulfur Diesel, Fatty Acid Methyl Esters and Select
Alternative Fuels
*Speaker: Joseph Suflita, University of Oklahoma*
Role of Free Radical Kinetics in Biocorrosion
*Speaker: Mark Maupin, Colorado School of Mines*
Studies on corrosion and biofouling detection and prevention using microelectrode arrays
*Speaker: Jason Ren, University of Colorado*
Corrosion of 1018 carbon steel in fuel/seawater incubations
*Speaker: Recep Avci, Montana State University*
Discussion
Keynote: Refueling Infrastructure-Status of Biofuels and Investigations
*Speaker: Kristy Moriarty, National Renewable Energy Laboratory*
Microbiologically Influenced Corrosion of Industrial Tank Materials during Exposure to Fuel-Grade Ethanol Environments
*Speaker: Jeff Sowards, National Institute of Standards and Technology*
What’s Going on Inside Today’s Fuel Storage Tank?
*Speaker: Lorri Grainawi, Steel Tank Institute*
Materials Susceptibility in Contaminated Alternative Fuel
*Speaker: Wendy Goodson, Air Force Research Laboratory*
Discussion, session wrap ups
**Day 2**
Keynote: Properties of Biodiesel and Other Biogenic Fuels
*Speaker: Gary Knothe, USDA*
Inevitable Changes in Measurements: Redefining What We Mean by “Fit-For-Purpose”
*Speaker: Tom Bruno, National Institute of Standards and Technology*
Overview of U. S. Biofuels Quality
*Speaker: Teresa Alleman, National Renewable Energy Laboratory*
Transcriptional Response and Adaptation of Bacteria to Jet Fuel
*Speaker: Oscar Ruiz, Air Force Research Laboratory*
Discussion
Conference Recap/Panel with Keynote speakers
Afternoon Tours at NIST
Hydrogen Embrittlement of Metals Facility – *Andrew Slifka*
Fuels – *Tom Bruno*
Microbiologically Influenced Corrosion and Mechanical Testing High Bay – *Jeff Sowards and Dash Weeks*
Environmental Microbiology – *Danielle France and Teresa Kirschling*
Precision Imaging Facility – *Ann Chiaramonti-Debay*

**Table 2:** Survey questionnaire

Questions for the Fuels Session
1. What prevents widespread alternative fuel use without fear of biocorrosion?
2. Are there any significant gaps in understanding the relationship of alternative fuels and biocorrosion? What do we not know about fuels and their relationships to biocorrosion?
3. What are the fundamental properties (properties that are directly related to theory) we need to understand to advance knowledge of biofuels?
4. What are the empirical (or fit for purpose) properties to understand to advance knowledge of biofuels?
5. Where do we need to relate empirical properties to fundamental properties to help in the field of biocorrosion?
6. What is the biggest challenge in the analysis of microbiologically contaminated fuels obtained from the field?
Questions for the Microbiology Session
7. What are the technical challenges/knowledge gaps in the relationship of microbes to biocorrosion?
8. To what extent do you feel understanding the microbial influences would advance the field of biocorrosion? Do you believe that identification of the type of microbe present, identification of microbial activity (acid producing, etc.) or just identifying that microbes are present is significant at this time? Why?
9. What would be the most significant advancement in microbial science that would allow for successful elimination or mitigation of biocorrosion?
10. How do you determine if corrosion is microbiologically influenced?
11. What microbes do you feel are most significant contributors to corrosion for your material issues?
Questions for the Infrastructure Session
12. What are the technical challenges/knowledge gaps in the relationship between infrastructure issues and biocorrosion?
13. What are the significant materials impacted by biocorrosion?
14. Can we quantify the current magnitude (cost, frequency) of biocorrosion in the US? How does this relate to the magnitude of corrosion generally? Will we be able to quantify how biocorrosion changes as a result of introduction of alternative fuels?
15. What are the current barriers to evaluating whether biocorrosion is an issue in a given situation?
a. What are the limitations of ASTM methods and field test kits in detecting/predicting biocorrosion in the field?
b. How can we increase the throughput and applicability of evaluation methods?
16. What are gaps in predicting when biocorrosion may occur and what impact it may have?
17. What metrics should be used to determine when mitigation strategies should be applied? Are there current needs for developing/implementing anti-fouling and anti-corrosion materials? What tests and metrics are appropriate for evaluating the efficacy of such materials?

## References

[1] Hays GF (2010). Now is the Time. Journal of Advanced Materials Research.

[2] Zhu XY, Lubeck J, Kilbane JJI (2003). Characterization of Microbial Communities in Gas Industry Pipelines. Applied and Environmental Microbiology.

[3] Beavers JA, Thompson NG External Corrosion of Oil and Natural Gas Pipelines. ASM Handbook, 2006. 13C, Corrosion.

[4] Shi X, Xie N, Gong J (2011). Recent Progress in the Research on Microbially Influenced Corrosion: A Bird’s Eye View through the Engineering Lens. Recent Patents on Corrosion Science.

[5] Muthukumar N (2003). Microbiologically influenced corrosion in petroleium product pipelines. Indian Journal of Experimental Biology.

[6] Lane RA (2005). Under the Microscope: Understanding, Detecting, and Preventing Microbiologically Influenced Corrosion. Journal of Failure Analysis and Prevention.

[7] Little BJ, Lee JS (2009). Microbiologically Influenced Corrosion. Kirk-Othmer Encyclopedia of Chemical Technology.

[8] Gu T (2012). New Understandings of Biocorrosion Mechanisms and their Classifications. J Microbial Biochem Technol.

[9] Cahn JW (2000). “Die Heterogenen Gleichgewichte (The Heterogeneous Equilibria)” a century later. Journal of Phase Equilibria.

[10] Striebich RC (2014). Characterization of the F-76 diesel and Jet-A aviation fuel hydrocarbon degradation profiles of Pseudomonas aeruginosa and Marinobacter hydrocarbonoclasticus. International Biodeterioration & Biodegradation.

[11] Aktas DF (2013). Effects of oxygen on biodegradation of fuels in a corroding environment. International Biodeterioration & Biodegradation.

[12] Zerda TW, Yuan X, Moore SM (2001). Effects of fuel additives on the microstructure of combustion engine deposites. Carbon.

[13] Yang F, Hanna MA, Sun R (2012). Value-added uses for crude glycerol - a byproduct of biodiesel production. Biotechnology for Biofuels.

[14] (2012). Detection, Testing, and Evaluation of Microbiologically Influenced Corrosion on Internal Surface of Pipelines. TM0212-2012.

[15] (1985). Biologically Induced Corrosion.

